# Low Back Exoskeletons in Industry 5.0: From Machines to Perceiving Co-Pilots—A State-of-the-Art Review

**DOI:** 10.3390/s25071958

**Published:** 2025-03-21

**Authors:** Andrea Dal Prete, Marta Gandolla, Giuseppe Andreoni, Francesco Braghin

**Affiliations:** 1 Mechanical Engineering Department, Politecnico di Milano, Via Giuseppe La Masa 1, 20156 Milan, Italy; marta.gandolla@polimi.it (M.G.); giuseppe.andreoni@polimi.it (G.A.); francesco.braghin@polimi.it (F.B.); 2 Bioengineering Laboratory, Scientific Institute, Bosisio Parini, 23842 Lecco, Italy

**Keywords:** back exoskeleton, lower-back pain, intelligent framework, wearable technology

## Abstract

This manuscript presents an updated review of back exoskeletons for occupational use, with a particular focus on sensor technology as a key enabler for intelligent and adaptive support. The study aims to identify key barriers to adoption and explore design characteristics which align these systems with the Industry 5.0 paradigm, where machines function as collaborative co-pilots alongside humans. We propose a structured design pipeline and analyze 32 exoskeletons across multiple dimensions, including design, actuation, control strategies, sensor networks, and intelligence. Additionally, we review eight simulation environments which support the early stages of exoskeleton development. Special emphasis is placed on sensor technology, highlighting its critical role in enhancing adaptability and intelligence. Our findings reveal that while 39.39% of exoskeletons accommodate asymmetric activities, kinematic compatibility remains a challenge. Furthermore, only 33.33% of the systems incorporated intelligent features, with just one being capable of adapting its response based on poor posture or real-time human–machine interaction feedback. The limited integration of advanced sensors and decision-making capabilities constrains their potential for dynamic and adaptive support. Open questions remain in high-level decision making, enhanced environmental awareness, and the development of generalizable methods for integrating sensor data into adaptive control strategies.

## 1. Introduction

**Figure 1:**
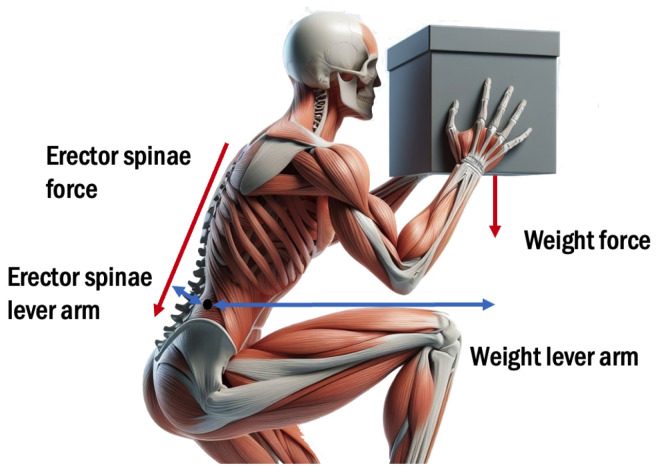
Equilibrium around the low back joint in the sagittal plane between the torques generated by the payload and the erector spinae muscles.

Despite advancements in automation, many industrial tasks still require human workers due to their superior decision making, precision, and versatility [1]. However, such activities, particularly manual material handling tasks like lifting and carrying, increase the risk of lower-back pain (LBP) and musculoskeletal disorders (MSDs) [2]. These tasks often lead to muscular fatigue and excessive loading on spinal joints (L4-L5 and L5-S1) [3,4], as the spinal column relies on complex interactions between muscles, tendons, and discs for stability. High compressive forces on the erector spinae muscles strongly contribute to these loads (Figure 1), potentially leading to long-term injuries such as intervertebral disc protrusion or prolapse [5,6]. In this context, back exoskeletons are being developed to reduce the risk of back injuries, with sensor technology playing a critical role in mitigating these issues by enabling real-time monitoring of spinal loads, muscular activity, and posture during physical tasks. By enhancing exoskeleton perception, sensors can support interventions aimed at reducing the risks associated with LBP and MSDs. Nevertheless, the prevalence of these disorders remains a significant concern, with 43% and 42% of European workers reporting back and upper limb pain, respectively, in 2017 [7].

### 1.1. The Role of Technology

In recent decades, there has been growing interest in collaborative and wearable solutions, such as back exoskeletons (BEs), to create safer working environments and reduce back injury risks. Despite advancements, the adoption of BEs in industry remains limited, prompting recent studies to investigate the critical factors for broader adoption [8,9]. While previous research examined technological advancements and potential future directions, this review focuses on the barriers to adoption and the key research questions which must be addressed to advance back exoskeleton development, with a particular emphasis on the role of sensors and perception. Notably, De Looze et al. [1] highlighted the potential of assistive exoskeletons to reduce physical loads in industrial settings, while Toxiri et al. [10] emphasized technological trends but overlooked novel actuation methods such as series elastic and twisted string actuators. Pérez et al. [11] and Xiloyannis et al. [12] addressed challenges in soft robotic suits, focusing on hip joint support, but paid limited attention to occupational back exoskeletons. Ali et al. [13] categorized existing exoskeletons by actuation type, structure, and assisted tasks, concluding that design choices impact comfort, biomechanical effectiveness, and cost. However, these reviews often neglect critical aspects such as early-stage design software, sensor networks, and embedded intelligence.

This review aligns with the Industry 5.0 paradigm, prioritizing seamless human–machine interaction to enhance safety and human capability [14]. In fact, while previous reviews primarily focused on the design of back exoskeletons, we argue that their successful industrial integration depends not only on advancements in lightweight, ergonomic, and kinematically compatible designs but also on significant improvements in perception and intelligence. This manuscript provides a comprehensive review of back exoskeletons, examining both design strategies and current control and perception capabilities. One key objective is to identify future research directions for enhancing high-level control and human–machine interaction. Unlike prior works, this review specifically explores emerging perception strategies and embedded intelligence to improve interaction fluidity. Furthermore, we propose validated simulation environments as a safer alternative for development, minimizing risks related to tests with subjects. Overall, we emphasize the crucial role of sensors, perception, and digital technologies in advancing the intelligence and adaptability of back exoskeletons. In summary, we propose a review to identify possible advancements in the digital perception and intelligent areas of back exoskeletons. Central to this review is the study of classical sensory systems and exploration of potential alternative sensors and perception systems which could enable real-time monitoring of human–machine interaction, a key aspect often overlooked in prior research. We examine technologies used in state-of-the-art solutions, identify the main needs and areas for improvement in exoskeletons, and propose potential solutions for future investigation.

### 1.2. Search Strategy

To compile the material for this review, we conducted a comprehensive search across multiple databases, including Scopus, PubMed, IEEE Xplore, and multidisciplinary research platforms such as Google Scholar. Additionally, we considered relevant records from other sources to ensure an inclusive selection. The inclusion and exclusion criteria for selecting the 32 exoskeletons were based on two key factors: (1) the solutions must have been developed within the last two decades, and (2) they must align with the thematic areas analyzed in this review. A similar approach was applied to the selection of simulation environments. To identify the relevant literature, we used a range of keywords, including but not limited to back exoskeleton, industrial exoskeleton, exoskeleton for occupational use, wearable robot, wearable sensor, wearable technology, back support device, industrial support device, exoskeleton intelligence, and exoskeleton smartness.

### 1.3. Paper Contribution and Structure

**Figure 2:**
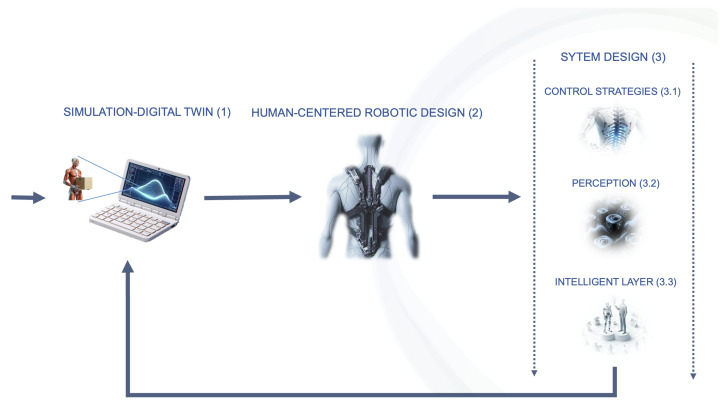
Back exoskeleton design pipeline and categories in the Industry 5.0 paradigm.

This review aims to analyze current back exoskeleton solutions beyond structural descriptions, emphasizing user-centered technology. Figure 2 outlines a potential design pipeline centered on the user, summarizing the key categories investigated.

This paper is organized as follows. Section 2 introduces simulation models and environments to aid in designing and assessing back exoskeletons’ impact on the user. Section 3 provides an overview of back support exoskeletons and explores design solutions addressing contemporary challenges. Section 4 delves into system design, focusing on (Section 4.1) control strategies for active exoskeletons to enhance human-robot interaction; (Section 4.2) user back monitoring via sensor networks for real-time perception of user-machine interaction; and (Section 4.3) an intelligent layer enabling adaptive responses based on sensor data. Finally, Section 5 and Section 6 discuss the current state of technology, propose future research directions, and present conclusions.

## 2. Simulation Environments for the Early Stages of Exoskeleton Development

Exoskeleton development often involves testing with subjects performing strenuous or unsafe tasks. However, within the Industry 5.0 paradigm, user-centered design begins early in the development process. Simulation environments have demonstrated their potential for deriving exoskeleton design specifications, enabling safer and more resource-efficient early-stage development [15], and simpler regression models derived from these simulations have been widely investigated. These tools, complemented by sensor technologies, could play a crucial role in accurately estimating spine loads, monitoring user interaction, and assessing task risks. These tools are collected in Table 1.

(1) The University of Michigan 3D Static Strength Prediction Program (3DSSPP™) [16] is a software tool for predicting the static strength for various postures, providing gender-specific force and compression data. It is effective for evaluating dangerous static postures in early exoskeleton designs but lacks dynamic analysis. Sensors could augment the 3DSSPP™ by supplying real-time kinematic inputs, enabling iterative testing of posture adjustments. (2) The Hand-Calculation Back Compressive Force (HCBCF) [17] is a simplified gender-specific back compression model which was compared across 6000 lifting tasks. While suitable for identifying high-risk tasks, it relies on predefined parameters and could benefit from wearable sensors for dynamically tracking torso flexion angles and upper-body mass. (3) The Linked-Segment Biomechanical Model (LSBM) [18] is a regression-based model for estimating L5-S1 compression forces during symmetric lifting. Despite its high accuracy, integration with exoskeletons requires sensor networks to capture real-time NIOSH horizontal and vertical data, trunk angles, and user loads. (4) Arjmand et al. derived regression models [19,20] from a finite element-based model for predicting spinal loads during symmetric and asymmetric lifting. Integrating this approach with sensor-based monitoring systems could provide a means to validate exoskeleton designs and ensure dynamic responses to user activities. (5) The simple polynomial equation (SPE) [21] estimates lower-back compression during 3D loading tasks while incorporating muscle co-activation. Even though it has been proven to be accurate, it would be challenging to embed within an exoskeleton without sensor-based inputs for dynamic adjustments. (6) The AnyBody modeling system [22] is advanced musculoskeletal simulation software which integrates external loads, including interaction with exoskeletons. Combined with force/torque sensors and motion capture systems, it can provide comprehensive analyses of user–exoskeleton interactions. (7) Similarly, OpenSim [23] is open-source software for musculoskeletal simulations with validated models for spinal loading [24]. Like AnyBody, OpenSim can integrate exoskeleton models and sensor inputs to simulate real-world scenarios. (8) Finally, the Santos Human Simulation Environment [25] is a digital human simulation platform incorporating AI and machine learning for predictive dynamics. Real-time data from smart sensors enhance its capacity to assess exoskeleton impacts on user movement and safety.

Although tools like AnyBody, OpenSim, and Santos are increasingly used during early-stage exoskeleton development [26,27], sensor technologies are essential for bridging the gap between simulation and real-world application. Wearable sensors such as inertial measurement units (IMUs), load cells, and EMG sensors provide continuous feedback on body kinematics, posture, and muscle activation, enabling more accurate simulations. In contrast, lightweight models like the HCBCF, LSBM, and SPE, which are derived from complex systems, could be embedded into exoskeletons for real-time detection of unsafe postures or tasks. However, this integration requires adapting these models to account for the presence of an exoskeleton, as well as implementing robust sensor networks to measure payload position, user dynamics, and posture—critical factors for estimating spinal loads. For example, force sensors embedded in exoskeleton joints can help monitor applied torque, while IMUs on the user’s body can capture angular displacements to improve load predictions [28]. Since all current models have been designed while assuming standard conditions without an exoskeleton, their adaptation to scenarios where users wear exoskeletons is essential. Continued development of simulation environments, combined with advanced measurement strategies, is expected to enhance exoskeleton safety and design strategies [29]. Overall, these advancements align with the goal of Industry 5.0 to create seamless, intelligent human-technology integration [30,31].

**Table 1:** Summary of simulation models and environments and their capabilities.

Simulation Model	Load Estimation	Joint	Planes or Dimensions	Model Inputs
3DSSPP™ [16]	compression, shear	L4-L5 and L5-S1	3D	body weight and height, payload position and weight, body posture
HCBCF [17]	compression	L4-L5	axial compression only	body weight and height, L5-S1 joint-hand distance, payload weight, and trunk sagittal flexion angle
LSBM [18]	compression	L5-S1	axial compression only	L5-S1 joint coordinates, subject weight, and handled weight magnitude or position
Regression models [19,20]	compression, shear	L5-S1	3D	sagittal trunk flexion, lumbopelvic ratio, payload and its anterior or lateral distance from L5-S1
SPE [21]	compression	L4-L5	axial compression only	flexion-extension, lateral bending, and axial twisting moment
AnyBody [22]	compression, shear, momentum	full body	3D	human body kinematics and dynamics and payload
OpenSim [23]	compression, shear, momentum	full body	3D	human body kinematics and dynamics and payload
Santos [25]	compression, shear, momentum	full body	3D	human body kinematics and dynamics and payload

## 3. Human-Centered Robotic Design

Human-centered design is pivotal in the development of back exoskeletons (BEs) [32]. While evidence shows that both rigid and soft BEs can reduce muscular activity [33,34], their impact on spinal loads remains underexplored due to the lack of reliable, noninvasive methods for direct measurement. Consequently, muscular activity reduction remains the primary metric for evaluating their effectiveness. Rigid exoskeleton models often struggle with tasks beyond their intended scope, potentially causing user discomfort and reducing acceptance [35]. These challenges highlight the importance of prioritizing user comfort, kinematic compatibility, and adaptability in exoskeleton design. Furthermore, past research showed the importance of sensor feedback to further enhance exoskeleton assistance [36]. Ensuring versatility driven by enhanced perception is critical to overcoming these barriers and achieving broader adoption within industry settings. In this context, we argue that a well-designed sensor network is crucial for enhancing exoskeleton effectiveness in both passive and active solutions. While exoskeletons are typically validated through EMG reduction, these evaluations often occur in controlled laboratory settings, raising concerns about their real-world applicability. To address this, real-time monitoring is essential for ensuring proper functionality and adapting (in the case of active solutions) the exoskeleton’s behavior based on the user’s current state of stress and workload. Although passive exoskeletons do not rely on feedback for control modulation, as they assist with passive mechanical components, an effective perception and monitoring layer remains valuable if not necessary. Such a system could track working conditions, verify proper support, or intervene if the user approaches dangerous regions, enhancing both safety and usability. A key limitation in real-time human back monitoring is the challenge of using EMG sensors in real-world settings. In this work, we explore alternative solutions for human back monitoring and propose a comprehensive investigation of viable approaches in Section 4.2. Additionally, we anticipate that the availability of a real-time monitoring layer would significantly accelerate research and development by enabling large-scale data collection in real working environments. This, in turn, would facilitate deeper analysis and drive further advancements in exoskeleton research.

### 3.1. Design and Actuation of Existing Passive Exoskeletons

Passive devices are generally more comfortable and lightweight than active exoskeletons, as they lack power sources and active components. These devices rely on mechanical deformation to store and release energy, redistributing forces to other joints and reducing spinal loads. However, their lack of a power supply often means they are not equipped with sensors, limiting their ability to perceive and adapt to user movements and external conditions. Incorporating sensors could significantly enhance the perception and adaptability of passive exoskeletons, enabling better integration with user dynamics. Most recent solutions are collected in Table 2.

For instance, the IX Back [37] uses springs or gas springs [52] for energy storage and release, while the Laevo v2 [41] incorporates a hybrid structure for flexibility. Soft solutions like LiftSuit 2.0 [43,44] and PLAD [45] use elastic bands to enhance fit and movement, while devices such as APEX [46], Smart Suit Lite [47], and B.A. Garment [48] further promote flexibility. Designs like SPEXOR [49] utilize flexible beams for passive actuation, and HULC [50] and VT-Lowe [51] employ hydraulic springs and carbon fiber beams for efficient energy transfer. Flexible exoskeletons reduce joint misalignment and allow for natural movement. However, their tight fit for support can cause discomfort and restrict motion, and their soft structures limit the ability to provide significant skeletal support. Rigid exoskeletons offer better muscular and skeletal support but compromise movement and adaptability, especially during tasks requiring dynamic or asymmetric motion.

### 3.2. Design and Actuation of Existing Active Exoskeletons

Table 3 summarizes current prototypes and commercial active back exoskeletons (BEs). Active BEs, equipped with batteries, control boards, actuators, and sensors, provide dynamic support but are often cumbersome. Similar to passive devices, they aim to redirect back forces to other joints, with active actuation theoretically offering superior back stress reduction for specific tasks, although this remains unproven [53]. Active devices excel in dynamic tasks compared with passive solutions, which are preferred for static activities [54]. Crucially, the inclusion of sensor technologies in active BEs enables intelligent monitoring and adaptable control strategies, broadening their application scope. Several prototypes, such as Hal [55], Apogee [56], and AWN-03 Panasonic [57], as well as Backbone [58], SIAT Waist Exo [59], and Hyundai H-WEX [60], rely on electric motors for torque generation. Innovations include parallel elastic actuators (PEAs), as seen in RoboMate [61], and series elastic actuators (SEAs), which are used in the spine-assistive exo [62] and APO [63]. These actuators integrate elastic components to enhance energy efficiency and adaptability to human kinematics [64]. Advanced designs, such as the Differential SEA (D-SEA) [33], enable balanced support during lifting tasks, though they may limit versatility. Further innovations include twisted string actuators (TSAs), lightweight and space-efficient systems combining rigid and elastic properties, as seen in the hip joint exoskeleton (HJE) [65] and soft suit [66]. Pneumatic actuators, mimicking back muscle behavior, are employed in devices like the Muscle Suit [67]. More flexible solutions have emerged to improve compatibility with human kinematics. Backbone [58] aligns with spinal displacement, and BSE [33] integrates human-like hip joint mobility. Spine-inspired designs, like those in [68], use linked vertebrae-like structures for natural spine simulation. Wearable suits like the Superflex Suit [69], ABX [70], and the suit in [34] employ TSAs or cable-driven motors for asymmetric lifting support, showing strong muscular activity reduction [34]. The SARE exosuit [71] exemplifies cutting-edge advancements by integrating smart textiles and soft sensors. Its multi-soft artificial muscles (MSAM) mimic spinal muscles, while the soft knitting sensor (SKS) monitors spine strain and curvature in real time. Such sensor technologies provide adaptive assistance and detailed biomechanical feedback, demonstrating the potential to revolutionize active BE functionality and the user experience. The Bilateral Back Extensor Exosuit (BBEX) mimics the human spine to assist with lifting tasks. It features a secondary erector spinae (SES) mechanism with vertebra-like modules connected by ball-and-socket joints and linear actuators, enabling flexion-extension, lateral bending, and axial rotation. The BBEX uses twisted elastic rotary-rail actuators (TERRAs) powered by brushless DC motors which are arranged bilaterally to replicate the erector spinae muscles, providing precise multi-degree-of-freedom (DoF) support for complex lifting tasks [72].

In general, rigid active BEs, such as Apogee, Hyundai H-WEX, APO, and BSE, are potentially more effective in supporting the muscular and skeletal structure of the back but are less kinematically compatible, which can lead to uneven load distribution and discomfort. On the other hand, soft solutions often provide a closer fit to the body, but there is uncertainty regarding their ability to support the skeletal structure of the back, particularly in relieving it from heavy loads and reducing the risk of spinal disc-related impairments.

## 4. System Design

### 4.1. Control Strategies of Active BE

Past research reviewed control strategies employed in active exoskeletons (BEs) [10], and we aim to enrich this exploration by categorizing (Table 4) active BEs based on sensor feedback, control strategies, and intelligence. Feedback for controlling exoskeleton support can come from physiological signals (e.g., sEMG) or kinematic and dynamic quantities like positions, velocities, accelerations (via IMUs), or ground reaction forces (via pressure insoles). The SIAT-WEXv2 exoskeleton uses IMUs and encoders to capture lumbar and leg angles and employs a novel control method which combines fuzzy adaptive algorithms with model-based control, allowing real-time adaptation to the wearer’s movements during activities like stoop lifting. The effectiveness of this system was assessed by measuring muscle activity reduction using sEMG [59]. Similarly, the Hyundai H-WEX focuses on the gluteus maximus, using a motor, hall sensor, and IMU to detect motion and provide assistive torque for activities like walking and lifting. This system was evaluated using sEMG, showing muscle activity reductions during specific tasks [60]. Robo-Mate employs electric motors and parallel elastic springs, with a torque sensor to measure the interaction torque. It integrates feedback from trunk inclination via an IMU and compensates for friction, improving torque control [61]. Another version of Robo-Mate used trunk acceleration-based control to improve torque control during fast movement transitions, aligning with user intentions and detecting weight holding via an arm-based Myo armband [73]. Zhang et al. designed a spine-assistive exoskeleton driven by an SEA powered by a brushless motor, incorporating various sensors for kinematic and kinetic data. This exoskeleton uses a hierarchical control system, with admittance control at the low level and assistive control at the high level. Its effectiveness in reducing muscle activity was evaluated using sEMG [62]. Lanotte et al. developed the APO exoskeleton with SEA and electromagnetic motors for trunk extension during lifting. This system uses real-time hip joint angle computation to detect intention and apply assistance, being validated through sEMG measurements during repetitive tasks [63]. Ding et al. developed a BSE featuring a differential series elastic actuator (D-SEA), with a controller which uses spring elongation feedback and hip joint angle and velocity data. Their system enabled smooth transitions between walking and lifting activities, reducing back muscle activation by 40% during lifting tasks without increasing leg muscle activity [33]. Barsomian et al. used impedance control in their exoskeleton, relying on motor encoders at the low back joint to optimize control and estimate external forces [74]. Yao et al. developed a biologically inspired soft suit using IMUs to monitor trunk flexion angles, motor adjustments, and transition detection during various movement phases, validated through muscular activity measurement with sEMG [66]. Yang et al.’s flexible, spine-inspired exoskeleton integrates torque sensors and IMUs for feedback during stoop lifting, using virtual impedance and PID control to regulate motor performance [68]. Molinaro et al. integrated load cells, IMUs, and ESCs for controlling an exosuit. Load cells measure shoulder attachment forces, while the IMU estimates trunk orientation. ESCs regulate actuator operation, adjusting assistance based on the user mode and cable tension. The effectiveness was evaluated using EMG and lumbar range of motion measurements [70]. Chung et al. investigated an exosuit using ribbon cables for symmetric and asymmetric lifting, driven by a high-torque density motor. It integrates three IMUs to measure kinematics and adjusts assistance based on the trunk angle and velocity via impedance control, resulting in muscle activity reduction and improved performance during a work simulation [34]. The BBEX control system integrates real-time posture estimation using a kinematic model and force/torque sensors, enabling the exosuit to dynamically modulate assistive forces based on the wearer’s movement. This ensures adaptive torque compensation during lifting, enhancing both efficiency and user comfort. Validation experiments conducted with 11 subjects demonstrated the effectiveness of the BBEX. Posture estimation achieved high accuracy, with an average error of approximately 22 mm. The exosuit successfully adjusted the assistive torque and forces for both symmetric and asymmetric lifts. In terms of physiological impact, BBEX reduced heart rates by up to 28.40% and perceived exertion by up to 10.70%, indicating lower physical strain. Additionally, it significantly lowered muscle fatigue, reducing erector spinae fatigue by up to 40.80%, and it decreased the L5-S1 compression force by up to 15.20%, alleviating spinal stress during lifting. Overall, the BBEX enhances spinal alignment, reduces muscle effort, and minimizes joint compression, making it a promising tool for occupational lifting support [72].

Despite the widespread use of sensors such as sEMG and IMUs for feedback, many active BEs still face challenges in achieving smooth transitions between control strategies and fully adapting to the varied needs of users. This lack of adaptability, especially in making real-time adjustments based on sensor data, limits the practical application of exoskeletons in dynamic, real-world environments. We suggest that future research should prioritize improving control strategies through better integration of sensor data to enhance adaptability, allowing exoskeletons to better cater to diverse user needs and boosting their overall effectiveness. Developing a robust sensor network, along with strategies for integrating feedback information to improve safety, monitoring, and user adaptability, is crucial. Moreover, significant gaps remain in methods for generalizing control strategies, estimating payloads, and monitoring spine loads or hazardous activities. Addressing these issues will be key to enabling exoskeletons to respond more adaptively, unlocking their full potential and improving safety and the user experience. Furthermore, many current systems lack the high-level control capabilities needed for environmental awareness and adaptive decision making, which could further enhance the utility and safety of exoskeletons in diverse applications.

### 4.2. Perception

In this review, we define BE perception as the capability to perceive user physiology, human–machine interaction, and kinematic information. Table 4 outlines the sensors used in each exoskeleton for both real-time feedback and validation. Both passive and active exoskeletons should aim to interpret user intentions effectively, facilitating unrestricted movement while providing significant assistance. Achieving this relies on the careful selection of sensors to gather pertinent data, which is challenging due to the complexity of understanding user intent. The underutilization of back exoskeletons in industry, despite research advancements, is partly attributed to their inability to offer real-time feedback on back stress levels and recognize users’ intentions for adaptability. Implementing a comprehensive sensor network across all powered exoskeletons is proposed as a valuable strategy to monitor user well-being and evaluate the exoskeleton’s impact, including muscular fatigue. With these two goals—user monitoring and intention detection—our research primarily focuses on two types of sensors: those capturing mechanical data related to user—exoskeleton interaction and those gathering physiological information. Mechanical sensors, such as IMUs [33,59,60,61,62,66,68], encoders [33,59,62,63], force/torque (motor current) sensors [59,60,61,62,63,66,68], and pressure sensors [62], are typically used for control purposes, aiding in human—machine interaction. In contrast, physiological sensors, primarily based on sEMG signals, monitor muscular activity to assess exoskeleton effectiveness, especially in back muscles like the erector spinae. These sensors were mainly used for final validation of back exoskeletons (BEs) [33,34,59,62,63,66]. Additionally, sEMG signals may inform control strategies, as seen in studies where arm-based sEMG detected load handling, adjusting the control accordingly [73]. Overall, the literature reveals that the sensors mentioned are primarily used for control purposes or post-design validation of exoskeletons. However, there is a growing need to utilize sensor networks to develop an intelligent layer on top of them. For example, Matijevich et al. [75] demonstrated that IMUs and pressure insoles offer effective data for estimating spine loads during lifting, suggesting their potential for monitoring spine stress. Despite this, such implementations have not occurred in back exoskeletons, as load weight and position are still necessary inputs and are challenging to estimate. Therefore, beyond control, these sensors could be valuable for indirectly monitoring user spine health. Simultaneously, electromyography is extensively used for measuring muscle effort and evaluating subject fatigue. However, its use requires expertise in placing surface electrodes on the skin to monitor target muscles, along with the need for skin preparation and cleaning protocols to ensure reliability. Impedance measurements between muscles and EMG electrodes can be affected by various factors, such as tissue composition, electrode-muscle distance, electrode size, and the presence of sweat, as discussed in prior research by Jung [76]. Hence, the characteristics of surface electromyography pose challenges in industrial settings, leading to its limited use for real-time feedback in control or monitoring, with its primary usage being for validation tests. To incorporate sensors on exoskeletons for monitoring back muscular activity and fatigue, innovative solutions to address sEMG limitations are necessary. Some viable alternatives to sEMG are summarized in Table 5. Near-infrared sensors (NIRSs) offer promising avenues for tracking muscular activity. The NIRS utilizes the increased concentration of hemoglobin, a molecule responsible for oxygen transport, in response to heightened muscle activity. This unique property enables the monitoring of blood flow using techniques like photoplethysmography and the modified Beer—Lambert law [77], leveraging the greater absorption index of hemoglobin in the near-infrared range [78]. Ultrasonography (USMG) could also be used to monitor muscular activity, as muscle activation generates ultrasound waves detectable by measurement systems. P.W. Hodges et al. [79] compared EMG and ultrasound sensor data for muscle activity, while X. Yang et al. [80] proposed a new ultrasound sensor architecture for prosthetic control applications. Furthermore, muscle activation, movement, and fatigue can be measured using mechanomyography (MMG). For instance, Tarata et al. [81] evaluated MMG’s feasibility in capturing muscle vibration and movement during contraction. While innovative, some of these solutions share a common drawback with sEMG; they require direct application to the user’s skin for optimal functionality. This limitation poses ergonomic challenges, preventing their integration into exoskeletons for continuous real-time monitoring. Embedding such sensors would require a technician to place them on the user each time they wear the exoskeleton, which is both economically and ergonomically impractical. On the other hand, biological yielding during muscle activation leads to heat dissipation, resulting in local increases in muscle and skin temperature, whic are detectable by infrared (IR) thermal cameras [82]. Studies by Hildebrandt et al. [83], Rodriguez-Sanz et al. [84], and Perpetuini et al. [85] demonstrated a correlation between muscular activity and injury with changes in skin temperature. Unlike other technologies, IR thermal cameras do not require direct contact with the skin; they might even be capable of gathering information through clothes. This would make them easily wearable without the need for expert supervision. Additionally, they are potentially non-sensitive to the presence of sweat. Possible drawbacks of thermal cameras include subject-specific body temperature variations, high thermal inertia (which can make temperature dynamics slower than muscular activity), and difficulty designing sensors for embedded applications with the required level of accuracy and precision. In this context, we believe thermal cameras for gathering insights about muscular activation deserve further investigation. Forcemyography (FMG) has also been investigated in past research to detect muscular activity correlated with volumetric changes due to muscle contraction [86,87]. The sensor is usually composed of several load cells connected to a bracelet-like structure and placed on the skin or even on clothes. It has proven correlation with muscular contraction, but past research shows that subject-specific anthropometric characteristics can strongly influence (by up to 23–30%) the output of the FMG signal acquisition [88,89]. FMG has been widely investigated for gesture recognition and upper-limb technologies [90], yet its integration into a back exoskeleton is still missing in the literature. In conclusion, each sensor provides an alternative opportunity to enhance exoskeleton perception. However, this category presents several open questions and a need for further research. The technologies employed in [72] seem particularly promising. Indeed, the BBEX incorporates an advanced sensor fusion system for real-time posture estimation, ensuring precise adaptation to the wearer’s movements. It employs inertial measurement units (IMUs), force/torque sensors, and strain gauges, which work together to track spinal alignment and joint loading during lifting tasks. By integrating kinematic modeling with these sensor inputs, BBEX achieves high-accuracy posture tracking, enabling the system to provide targeted assistance while maintaining natural movement patterns. We believe that these types of sensory feedback are necessary to advance exoskeleton perception and effectiveness.

### 4.3. Intelligent Layer

As discussed in Section 4.1, BEs generally lack adaptability across different working scenarios. Some exoskeletons, such as the IX-Back, include an activity recognition layer without sensors [37], while others, like the Hyundai H-WE, APO, and Chang et al.’s exosuit, use activity or lifting phase recognition based on thresholds (e.g., angles), rather than leveraging machine learning for generalization. The SIAT-WEXv2, in addition to activity recognition, incorporates an adaptive fuzzy-based control strategy which adjusts based on the trunk tilt and hip joint flexion angles. Similarly, the BSE [33] detects whether the user is lifting or walking, adapting its assistive strategy using a sigmoid function for smooth control transitions. Robo-Mate employs an EMG-based Myo armband to detect if the user is holding weight [73], while Zhang et al.’s spine-assistive exoskeleton uses gloves with pressure sensors to detect weight holding. Yao et al.’s soft suit uses an IMU to detect four phases of lifting activities and generate a reference torque path accordingly [66]. More advanced systems, like the Seismic Suit, incorporate on-suit sensors and processors to analyze postures and activities, enabling continuous improvement over time via IoT data reporting [69]. Apogee integrates an AI-driven safety layer for posture correction, adapting to user needs and providing real-time alerts for poor posture or incorrect lifting. It also connects to industrial IoT platforms for seamless integration into smart factory ecosystems [56]. Recent advancements in posture estimation and adaptive intelligent control were proposed in [72], particularly with the BBEX, which integrates an adaptive control system capable of dynamically modulating assistance based on real-time user movement and external forces. Utilizing a kinematic model, the system continuously adjusts assistive torques and forces to account for changes in lifting asymmetry, posture deviations, and varying task demands. Furthermore, the exosuit incorporates machine learning optimization, enabling it to learn from user behavior and progressively refine its control strategies to enhance both comfort and efficiency. This intelligent, adaptive torque compensation ensures seamless integration with the wearer’s biomechanics, improving stability and ergonomic support during dynamic lifting tasks. Despite these advancements, exoskeletons generally remain limited in terms of intelligence, relying on predefined triggers and smooth transitions between a small set of activities. They lack high-level decision-making abilities which would allow them to better adapt to users’ needs and environmental factors. For example, if exoskeletons could assess factors such as payload, back strain, activity history, and future intentions, then they could better modulate support in real time. Research has explored methods for payload estimation [28,73], yet challenges remain, particularly in reducing latency to anticipate weight before lifting begins. Since the most significant strain on the back occurs early in the lifting cycle [91], preemptive weight assessment could enhance assistance. These advancements could transform exoskeletons from static machines to dynamic, active partners in user efforts. We advocate for further investigation into machine learning techniques to improve decision making and enhance the adaptability of exoskeletons, shifting them from mere tools to responsive, intelligent systems.

## 5. Results and Discussion

### 5.1. Results

**Figure 3:**
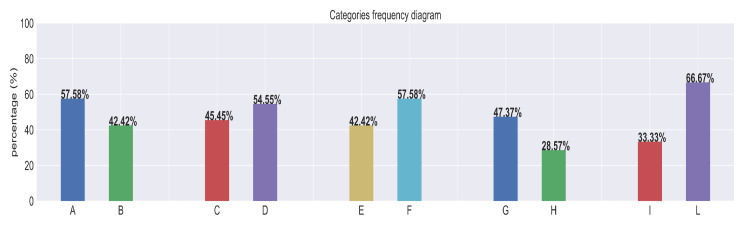
Frequencies diagram. Categories: A = percentage of active exo, B = percentage of passive exo, C = percentage of commercial solutions, D = percentage of prototypes, E = percentage of soft solutions, F = percentage of rigid solutions, G = percentage of solutions providing asymmetric support among actives, H = percentage of solutions providing asymmetric support among passives, I = percentage of solutions employing at least one intelligent feature, L = percentage of solutions not employing any intelligent feature.

In this review, a total of 32 exoskeletons were analyzed. Figure 3 collects the frequencies of the categories which were considered. In particular, among all the solutions that were collected, 57.58% were active (category A), and 45.45% were commercial solutions (category C); 42.42% were soft solutions (category E). Overall, 47.37% of the active solutions (category G) and 28.57% of the passive ones (category H), for 39.39% of the total, were considered able to provide asymmetric support and avoid user movement constraints. As a metric to assess whether an exoskeleton can provide asymmetric assistance, we considered whether it had been successfully tested in experimental settings or conceptually designed with the necessary degrees of freedom and range of motion to support such functionality. Finally, only 33.33% of the solutions employed at least one intelligent feature (category I), including those which could just distinguish between lifting and walking. Nevertheless, just two solutions (10.52% of the active solutions and 6.06% of the total) employed a full intelligent layer to monitor the user’s back and adapt to the current situation (Apogee and BBEX). In this manuscript, we considered an intelligent feature as a high-level control mechanism that leverages sensor feedback to adapt low-level control strategies based on user intent, environmental perception, or other contextual factors [92]. Given our focus on intelligence, we specifically classified as intelligent only those high-level features which incorporate machine learning techniques.

### 5.2. Discussion

The results show that previous research on back exoskeletons explored various kinematically compatible designs, including both rigid and soft solutions. Despite their potential, these solutions each present distinct challenges in terms of functionality and user comfort. While soft solutions offer better body fit and muscular support, rigid ones may be more suitable for spine support, particularly under compressive loads. A comprehensive ergonomic design should offer support for both muscular structures during lifting tasks and a rigid structure for transmitting forces from the spine to other joints while ensuring user comfort, as shown by Kim et al. [72]. However, the capacity of current solutions to support asymmetric activities remains limited. Although active solutions are generally more adaptable, with 47.37% supporting asymmetric activities, but there are still significant constraints on user movement, particularly in passive designs, where only 28.57% of solutions provide the necessary support. This highlights a fundamental limitation in current designs which restricts the ranges of movement and activities that users can perform effectively. There is a critical need for further research into control strategies which can better accommodate such activities, ensuring smoother and more intuitive user experiences. Approximately 33.33% of active exoskeletons incorporate a recognition layer, which is intended to identify user activities or lifting-related patterns. However, many of these systems either recognize too few activities or lack smooth control switching, leading to instability and discomfort during use. None of the solutions investigated incorporate technology to estimate the payload, which is in part because the current investigated technologies are limited to foot load cells, which make the exoskeleton bulkier. Although some researchers have explored more ergonomic IMU-based sensors for payload estimation [28], these require further validation when embedded in exoskeletons, and issues such as latency remain unresolved. These unresolved latency problems severely limit the practical effectiveness of exoskeletons in real-time applications. In this context, we argue that greater integration of machine learning and computer vision techniques could significantly enhance the adaptability and smoothness of control strategies. These technologies could help overcome the limitations posed by current sensor technologies, especially in addressing the latency problem. For example, computer vision-based techniques could remotely infer payload information, which could help mitigate latency issues and address subject-specific variability, making exoskeletons more adaptable to diverse real-world conditions. However, this approach presents new challenges, particularly when the lifted objects are not directly visible (e.g., lifting boxes with unknown contents). In such cases, sensor fusion with recently developed techniques may offer a promising approach to improving estimation accuracy and reliability. Non-contact sensor technologies, such as thermal sensors for muscular activity monitoring, also present promising opportunities to enhance sensor networks, enabling more comprehensive monitoring of both user movements and back muscle activity. Nevertheless, there is insufficient research exploring these sensor technologies in the context of exoskeletons, and further investigation is necessary to assess their effectiveness. Among the investigated back exoskeletons, only Apogee employs an intelligent layer for real-time health monitoring and response adaptation, highlighting the critical need for more advanced intelligent layers to accelerate the development and validation of exoskeletons. However, as was introduced in Section 4.2, a proper sensor network is needed to gain data directly related to the user activity and build an intelligent layer on top of it. Therefore, we emphasize the need for further research into sensor networks capable of providing real-time contextual data for intelligent control. In terms of simulation environments, we argue that their expanded use and further investigation could unlock the full potential of exoskeleton development. This would not only enhance the accuracy and efficiency of the design process but also enable more refined high-level control strategies. Previous research has demonstrated that, particularly in early-stage design [15], simulation environments can accelerate convergence toward effective design solutions while reducing the need for human subject testing, thereby minimizing the risk of overloading and injuries. Moreover, while simulation environments have already been increasingly employed in other domains, such as lower-limb exoskeletons, to develop intelligent high-level control strategies [26,27], they have also led to significant performance improvements in final implementations. Drawing a parallel with reinforcement learning advancements (particularly in humanoid robotics), where the availability of high-quality simulation environments has proven crucial for achieving strong results [93,94,95], we propose that further integration of simulation tools could unlock the full potential of back exoskeleton development. By enabling more efficient design processes and reducing the need for extensive human subject testing, simulations can minimize the risks of overloading and injury during the design phase. Additionally, a holistic approach to exoskeleton development must consider not only technical performance but also ethical implications, user feedback, and international perspectives. User feedback plays a critical role in refining the design and functionality of exoskeletons, ensuring they effectively address the real-world needs of users, as highlighted in previous studies [96,97]. Ethical considerations, including privacy, consent, and the impact on labor markets, must be addressed to promote responsible use and foster widespread acceptance of wearable technologies. An international perspective is also crucial for adapting exoskeletons to different cultural, regulatory, and economic contexts, which will influence their global adoption. While real-world data collection for high-level control models presents privacy and safety concerns, simulation environments provide a valuable alternative by allowing the generation of synthetic data which closely mimic real-world conditions. This approach enables the development of robust models without the need for invasive data collection, ensuring personal privacy while optimizing exoskeleton performance across diverse scenarios. We speculate that integrating these factors into exoskeleton development will enhance their practical applicability, foster ethical innovation, and support their successful deployment in varied contexts.

## 6. Conclusions

Recent advancements in back exoskeletons have focused on improving kinematic compatibility, monitoring, and control strategies. These systems now incorporate lightweight designs while maintaining or enhancing support capabilities. Emerging solutions prioritize human–exoskeleton interaction, with early-stage software and lighter models for back monitoring becoming increasingly prevalent. Researchers are exploring strategies for real-time payload estimation, muscular activity monitoring, and spine load assessment. From a control perspective, exoskeletons ranging from soft to rigid and passive to active designs have demonstrated efficacy in reducing back muscular activity without increasing the load on other joints or muscles. Advances in sensor technologies have improved the collection of kinematic, dynamic, interaction, and physiological data.

However, significant challenges remain. Most exoskeletons lack universal effectiveness across diverse work scenarios, particularly in ensuring safety, comfort, and adaptability to dynamic activities. Functional performance validation standards are critical to address these limitations [98]. Both active and passive exoskeletons currently lack intelligent monitoring layers capable of real-time back health assessment. In particular, unresolved questions include how to accurately estimate payloads, infer real-time muscular stress and spine load conditions, and utilize these insights as feedback for adaptive control. Such capabilities could optimize exoskeleton performance, improving efficacy during demanding tasks while minimizing unnecessary use. Moreover, comprehensive data collection would further advance research and development, promoting broader adoption of back exoskeletons in industrial applications.

The primary concerns regarding industrial back exoskeletons include discomfort, limited adaptability to dynamic environments, and challenges in usability. Addressing these requires systems which combine soft and rigid design elements to improve kinematic compatibility while retaining the benefits of both approaches. Integrating an advanced sensor network for enhanced perception and environmental understanding is crucial. Future efforts should focus on developing intelligent algorithms to extract actionable insights from sensor data. Such systems could enable exoskeletons to adapt to real-time conditions, maximizing their impact on user support and safety. Ultimately, the goal is to transition from mere support machines to collaborative agents, namely co-pilots which share the user’s workload and enhance their overall performance.

## Figures and Tables

**Table 2:** Passive exoskeleton. S = symmetric, AS = asymmetric. P = prototype, C = commercial.

EXO ID	TASK	STRUCTURE	ACTUATION	P or C
IX-BACK [37]	stoop or squat S or AS lifting	rigid	springs	C
Comau MateXB [38]	stoop or squat lifting	rigid	springs	C
Aldak [39]	stoop or squat lifting	rigid	springs	C
- [40]	stoop or squat lifting	rigid	springs	P
Laevo v2 [41]	S or AS stoop or squat lifting	hybrid	gas springs	C
IX BACK (AIR) [42]	stoop or squat lifting	rigid	gas springs	C
LiftSuit 2.0 [43,44]	stoop lifting	soft	textile springs	C
PLAD [45]	stoop or squat lifting, holding	soft	elastic bands	P
APEX [46]	S or AS stoop lifting	soft	elastic bands	C
Smart Suit Lite [47]	stoop lifting	soft	elastic bands	P
B.A. Garment [48]	stoop or squat lifting	soft	elastic bands	P
SPEXOR [49]	stoop or squat lifting	flexible	flexible beams	P
HULC [50]	lifting	rigid	hydraulic	C
VT-Lowe exoskeleton [51]	S or AS stoop or squat and freestyle	rigid	carbon fiber beams	P or C

**Table 3:** Active exoskeleton. S = symmetric, AS = asymmetric. P = prototype, C = commercial.

EXO ID	TASK	STRUCTURE	ACTUATION	P/C
Hal [55]	S or AS lifting	rigid	electric motor	C
Apogee [56]	stoop or squat lifting, carrying	rigid	electric motor	C
AWN-03 Panasonic [57]	stoop or squat lifting	rigid	electric motor	C
SIAT WEXv2 [59]	stoop or squat lifting	rigid	electric motor	P
Hyundai H-WEX [60]	lifting	rigid	electric motor, wires	P
RoboMate [61]	stoop or squat lifting	rigid	PEAs	P
Spine-assistive exo [62]	S or AS stoop or squat lifting	rigid	SEAs	P
APO [63]	stoop lifting	rigid	SEAs	P
BSE [33]	stoop or squat	rigid	D-SEA	P
HJE [65]	stoop or squat lifting	rigid	TSA	P
Backbone [58]	squat or stoop lifting	rigid	electric motor	P
Soft suit [66]	S or AS stoop lifting	soft	TSA	P
Muscle Suit [67]	S or AS lifting	soft	pneumatic (compressed air)	C
Spine-inspired [68]	S or AS stoop lifting	soft	electric motors, cable driven	P
Superflex [69]	stoop or squat lifting	soft	electric muscular actuators	P
ABX [70]	S or AS stoop or squat lifting	soft	motor plus cable	P
Active back exosuit [34]	S or AS stoop or squat lifting, carrying	soft	motor plus ribbon cable	P or C
SARE [71]	stoop lifting	soft	MSAM	P
BBEX [72]	S or AS stoop or squat lifting	hybrid	secondary erector spinae (SES) mechanism	P

**Table 4:** Active exoskeleton control categorized by sensors, control feedback, and control strategy.

EXO ID	Kinematic or Dynamic Sensors	Physiological Sensors	Control Feedback	Control Strategy
SIAT-WEXv2	IMU, encoders	sEMG for validation only (10 subjects)	Human–machine movement disparities, hip angle	Model-based control and fuzzy adaptive algorithm
Hyundai H-WE	IMU, Hall sensor	sEMG for validation only (9 subjects)	Upper body absolute sagittal inclination	Friction and gravity compensation, virtual spring, feedback, activity recognition
Robo-Mate	Torque sensor, IMU	sEMG for control	Human–machine interaction torque, trunk inclination, acceleration	Torque feedforward and feedback PD control based on trunk inclination, friction compensation, acceleration based-control
Spine-assistive	Encoders, IMUs, strain gauge, pressure sensor	sEMG for validation only (1 subject)	Position, velocity, acceleration, interaction forces, holding pressure	Torque feedforward and feedback PD control based on trunk inclination, friction compensation, acceleration based-control
APO	Encoders	sEMG for validation only (5 subjects)	Hip angle and velocity	Reference torque bell-shaped trajectory control
BSE	Longitudinal encoder, encoder, IMU	sEMG for validation only (14 subjects)	Hip angle and velocity	Virtual impedance, feedforward and feedback torque control
Soft suit	IMU, force sensor	sEMG for validation only (1 subject)	Tensile force, trunk flexion angle, velocity acceleration	Force control
Spine-inspired	Torque sensor, IMU	-	Interaction force, trunk motion	Virtual impedance force reference PID control. PID velocity and current low-level control
ABX	Load cells, IMU	sEMG for validation only	Interaction force, trunk orientation	Tension force control
Active back exosuit	IMUs, Load cells	sEMG for validation only (15 subjects)	Relative trunk angle and velocity	Adaptive impedance control
Backbone	Encoder	-	Reference trajectory	Impedance & LQR state-space control
BBEX [72]	Force/torque as well as kinematic sensors	-	Posture estimation feedback, force/torque feedback	P

**Table 5:** Possible solutions to replace electromyographic sensors. NIRS = near-infrared sensor, USMG = ultrasonography, MMG = mechanomyography, EMG = electromyography, IRT = infrared thermal camera and sensor.

Sensor Type	Reliability	Correlation to Muscular Activity	Need to Be Placed on the Skin	Inertia	Sensitiveness to Artifacts
NIRS	good	high	yes	moderate	modearte
USMG	good	high	yes	low	modearte
MMG	good	very high	yes	low	high
EMG	good or high	very high	yes	low	high
IRT	good	potentially moderate	no	moderate	moderate
FMG	good	high	no	low	moderate

## Data Availability

Data are contained within the article.

## References

[1] De Looze M.P., Bosch T., Krause F., Stadler K.S., O’Sullivan L.W. (2016). Exoskeletons for industrial application and their potential effects on physical work load. Ergonomics.

[2] Bernard B.P., Putz-Anderson V. (1997). Musculoskeletal Disorders and Workplace Factors: A Critical Review of Epidemiologic Evidence for Work-Related Musculoskeletal Disorders of the Neck, Upper Extremity, and Low Back.

[3] Dolan P., Adams M.A. (1998). Repetitive lifting tasks fatigue the back muscles and increase the bending moment acting on the lumbar spine. J. Biomech..

[4] Van der Have A., Van Rossom S., Jonkers I. (2019). Squat lifting imposes higher peak joint and muscle loading compared to stoop lifting. Appl. Sci..

[5] Kingma I., Faber G.S., van Dieën J.H. (2010). How to lift a box that is too large to fit between the knees. Ergonomics.

[6] Berger-Roscher N., Casaroli G., Rasche V., Villa T., Galbusera F., Wilke H.-J. (2017). Influence of complex loading conditions on intervertebral disc failure. Spine.

[7] Parent-Thirion A., Biletta I., Cabrita J. (2017). Sixth European Working Conditions Survey—Overview Report.

[8] Gonsalves N., Akanmu A., Shojaei A., Agee P. (2024). Factors influencing the adoption of passive exoskeletons in the construction industry: Industry perspectives. Int. J. Ind. Ergon..

[9] Crea S., Beckerle P., De Looze M., De Pauw K., Grazi L., Kermavnar T., Masood J., O’sullivan L.W., Pacifico I., Rodriguez-Guerrero C. (2021). Occupational exoskeletons: A roadmap toward large-scale adoption. Methodology and challenges of bringing exoskeletons to workplaces. Wearable Technol..

[10] Toxiri S., Näf M.B., Lazzaroni M., Fernández J., Sposito M., Poliero T., Monica L., Anastasi S., Caldwell D.G., Ortiz J. (2019). Back-support exoskeletons for occupational use: An overview of technological advances and trends. IISE Trans. Occup. Ergon. Hum. Factors.

[11] Pérez Vidal A.F., Morales J.Y.R., Torres G.O., Vázquez F.d.J.S., Rojas A.C., Mendoza J.A.B., Cerda J.C.R. (2021). Soft exoskeletons: Development, requirements, and challenges of the last decade. Actuators.

[12] Xiloyannis M., Alicea R., Georgarakis A.-M., Haufe F.L., Wolf P., Masia L., Riener R. (2021). Soft robotic suits: State of the art, core technologies, and open challenges. IEEE Trans. Robot..

[13] Ali A., Fontanari V., Schmoelz W., Agrawal S.K. (2021). Systematic review of back-support exoskeletons and soft robotic suits. Front. Bioeng. Biotechnol..

[14] Breque M., De Nul L., Petridis A. (2021). European Commission, Directorate-General for Research and Innovation, Industry 5.0—Towards a Sustainable, Human-Centric, and Resilient European Industry.

[15] Becattini N., Patriarca L., Scaccabarozzi DParenti P., Dal Prete A., Gandolla M. (2024). An integrated survey-simulation approach for exoskeleton performance estimation. Proc. Des. Soc..

[16] 3DSSPP™. https://c4e.engin.umich.edu/tools-services/3dsspp-software/.

[17] Merryweather A.S., Loertscher M.C., Bloswick D.S. (2009). A Revised Back Compressive Force Estimation Model for Ergonomic Evaluation of Lifting Tasks. Work.

[18] Potvin J.R. (1997). Use of NIOSH equation inputs to calculate lumbosacral compression forces. Ergonomics.

[19] Arjmand N., Plamondon A., Shirazi-Adl A., Larivière C., Parnianpour M. (2011). Predictive equations to estimate spinal loads in symmetric lifting tasks. J. Biomech..

[20] Arjmand N., Plamondon A., Shirazi-Adl A., Parnianpour M., Larivière C. (2012). Predictive equations for lumbar spine loads in load-dependent asymmetric one- and two-handed lifting activities. Clin. Biomech..

[21] McGill S.M., Norman R.W., Cholewicki J. (1996). A simple polynomial that predicts low-back compression during complex 3-D tasks. Ergonomics.

[22] Damsgaard M., Rasmussen J., Christensen S.T., Surma E., De Zee M. (2006). Analysis of musculoskeletal systems in the AnyBody modeling system. Simul. Model. Pract. Theory.

[23] Delp S.L., Anderson F.C., Arnold A.S., Loan P., Habib A., John C.T., Guendelman E., Thelen D.G. (2007). OpenSim: Open-source software to create and analyze dynamic simulations of movement. IEEE Trans. Biomed. Eng..

[24] Bruno A.G., Bouxsein M.L., Anderson D.E. (2015). Development and validation of a musculoskeletal model of the fully articulated thoracolumbar spine and rib cage. J. Biobech. Eng..

[25] Abdel-Malek K., Arora J., Bhatt R., Farrell K., Murphy C., Kregel K. (2019). Chapter 6—Santos: An Integrated Human Modeling and Simulation Platform.

[26] Molinaro D.D., Scherpereel K.L., Schonhaut E.B., Evangelopoulos G., Shepherd M.K., Young A.J. (2024). Task-agnostic exoskeleton control via biological joint moment estimation. Nature.

[27] Luo S., Jiang M., Zhang S., Zhu J., Yu S., Silva I.D., Wang T., Rouse E., Zhou B., Yuk H. (2024). Experiment-free exoskeleton assistance via learning in simulation. Nature.

[28] Pesenti M., Invernizzi G., Mazzella J., Bocciolone M., Pedrocchi A., Gandolla M. (2023). IMU-based human activity recognition and payload classification for low-back exoskeletons. Sci. Rep..

[29] Bianco N.A., Collins S.H., Liu K., Delp S.L. (2023). Simulating the effect of ankle plantarflexion and inversion-eversion exoskeleton torques on center of mass kinematics during walking. PLoS Comput. Biol..

[30] Beaucage-Gauvreau E., Robertson W.S.P., Brandon S.C.E., Fraser R., Freeman B.J.C., Graham R.B., Thewlis D., Jones C.F. (2019). Validation of an OpenSim full-body model with detailed lumbar spine for estimating lower lumbar spine loads during symmetric and asymmetric lifting tasks. Comput. Methods Biomech. Biomed. Eng..

[31] Rajaee M.A., Arjmand N., Shirazi-Adl A., Plamondon A., Schmidt H. (2015). Comparative evaluation of six quantitative lifting tools to estimate spine loads during static activities. Appl. Ergon..

[32] Kermavnar T., de Vries A.W., de Looze M.P., O’sullivan L.W. (2021). Effects of industrial back-support exoskeletons on body loading and user experience: An updated systematic review. Ergonomics.

[33] Ding S., Reyes F.A., Bhattacharya S., Narayan A., Han S., Seyram O., Yu H. (2024). A Novel Back-Support Exoskeleton With a Differential Series Elastic Actuator for Lifting Assistance. IEEE Trans. Robot..

[34] Chung J., Quirk D.A., Applegate M., Rouleau M., Degenhardt N., Galiana I., Dalton D., Awad L.N., Walsh C.J. (2024). Lightweight active back exosuit reduces muscular effort during an hour-long order picking task. Commun. Eng..

[35] Madinei S., Nussbaum M.A. (2023). Estimating lumbar spine loading when using back-support exoskeletons in lifting tasks. J. Biomech..

[36] De Miguel-Fernández J., Salazar-Del Rio M., Rey-Prieto M., Bayón C., Guirao-Cano L., Font-Llagunes J.M., Lobo-Prat J. (2023). Inertial sensors for gait monitoring and design of adaptive controllers for exoskeletons after stroke: A feasibility study. Front. Bioeng. Biotechnol..

[37] (2020). Suitx. https://www.suitx.com/.

[38] (2023). Comau Mate. https://mate.comau.com.

[39] (2023). Aldak. https://exoskeletonreport.com/product/aldak-passive/.

[40] Li P.L., Achiche S., Blanchet L., Lecours S., Raison M. (2019). Design of an Assistive Trunk Exoskeleton Based on Multibody Dynamic Modelling. arXiv.

[41] (2016). Laevo v2. https://exoskeletonreport.com/product/laevo/.

[42] (2023). Backx. https://exoskeletonreport.com/product/backx/.

[43] Van Sluijs R., Scholtysik T., Brunner A., Kuoni L., Bee D., Kos M., Bartenbach V., Lambercy O. (2024). Design and evaluation of the OmniSuit: A passive occupational exoskeleton for back and shoulder support. Appl. Ergon..

[44] Van Sluijs R.M., Wehrli M., Brunner A., Lambercy O. (2023). Evaluation of the physiological benefits of a passive back-support exoskeleton during lifting and working in forward leaning postures. J. Biomech..

[45] Whitfield B.H., Costigan P.A., Stevenson J.M., Smallman C.L. (2014). Effect of an on-body ergonomic aid on oxygen consumption during a repetitive lifting task. Int. J. Ind. Ergon..

[46] Herowear (2020). APEX Exosuit. https://herowearexo.com/.

[47] Imamura Y., Tanaka T., Tanaka T., Suzuki Y., Takizawa K., Yamanaka M. (2014). Analysis of Trunk Stabilization Effect by Passive Power-Assist Device. J. Robot. Mechatronics.

[48] Lamers E.P., Yang A.J., Zelik K.E. (2018). Feasibility of a Biomechanically-Assistive Garment to Reduce Low Back Loading During Leaning and Lifting. IEEE Trans. Biomed. Eng..

[49] Näf M.B., Koopman A.S., Baltrusch S., Rodriguez-Guerrero C., Vanderborght B., Lefeber D. (2018). Passive Back Support Exoskeleton Improves Range of Motion Using Flexible Beams. Front. Robot. AI.

[50] (2020). Exobionics. https://eksobionics.com/eksoworks/.

[51] Alemi M.M., Geissinger J., Simon A.A., Chang S.E., Asbeck A.T. (2019). A passive exoskeleton reduces peak and mean EMG during symmetric and asymmetric lifting. J. Electromyogr. Kinesiol..

[52] Kornhauser A.A. (1994). Dynamic Modeling of Gas Springs. J. Dyn. Syst. Meas. Control.

[53] Govaerts R., De Bock S., Provyn S., Vanderborght B., Roelands B., Meeusen R., De Pauw K. (2023). The impact of an active and passive industrial back exoskeleton on functional performance. Ergonomics.

[54] Poliero T., Fanti V., Sposito M., Caldwell D.G., Natali C.D. (2022). Active and Passive Back-Support Exoskeletons: A Comparison in Static and Dynamic Tasks. IEEE Robot. Autom. Lett..

[55] Hal Lumbar Support. https://www.cyberdyne.jp/english/products/Lumbar_LaborSupport.html.

[56] (2023). Apogee. https://germanbionic.com/en/solutions/exoskeletons/apogee/.

[57] Li R.Y.M., Ng D.P.L. (2018). Wearable Robotics, Industrial Robots and Construction Worker’s Safety and Health. Advances in Human Factors in Robots and Unmanned Systems.

[58] Roveda L., Savani L., Arlati S., Dinon T., Legnani G., Molinari Tosatti L. (2020). Design methodology of an active back-support exoskeleton with adaptable backbone-based kinematics. Int. J. Ind. Ergon..

[59] Ji X., Wang D., Li P., Zheng L., Sun J., Wu X. (2020). SIAT-WEXv2: A Wearable Exoskeleton for Reducing Lumbar Load during Lifting Tasks. Complexity.

[60] Ko H.K., Lee S.W., Koo D.H., Lee I., Hyun D.J. (2018). Waist-assistive exoskeleton powered by a singular actuation mechanism for prevention of back-injury. Robot. Auton. Syst..

[61] Toxiri S., Calanca A., Ortiz J., Fiorini P., Caldwell D.G. (2018). A Parallel-Elastic Actuator for a Torque-Controlled Back-Support Exoskeleton. IEEE Robot. Autom. Lett..

[62] Zhang T., Huang H. (2018). A Lower-Back Robotic Exoskeleton: Industrial Handling Augmentation Used to Provide Spinal Support. IEEE Robot. Autom. Mag..

[63] Lanotte F., Grazi L., Chen B., Vitiello N., Crea S. A Low-Back Exoskeleton can Reduce the Erector Spinae Muscles Activity During Freestyle Symmetrical Load Lifting Tasks. Proceedings of the 7th IEEE International Conference on Biomedical Robotics and Biomechatronics (Biorob).

[64] Jafari A., Tsagarakis N., Caldwell D. (2015). Energy efficient actuators with adjustable stiffness: A review on AwAS, AwAS-II and CompACT VSA changing stiffness based on lever mechanism. Ind. Robot..

[65] Seong H.S., Kim D.H., Gaponov I., Ryu J.H. Development of a Twisted String Actuator-based Exoskeleton for Hip Joint Assistance in Lifting Tasks. Proceedings of the IEEE International Conference on Robotics and Automation (ICRA).

[66] Yao Z., Linnenberg C., Weidner R., Wulfsberg J. Development of A Soft Power Suit for Lower Back Assistance. Proceedings of the International Conference on Robotics and Automation (ICRA).

[67] (2023). Muscle Suit. https://exoskeletonreport.com/product/muscle-suit/.

[68] Yang X., Huang T.-H., Hu H., Yu S., Zhang S., Zhou X., Carriero A., Yue G., Su H. (2019). Spine-inspired continuum soft exoskeleton for Stoop lifting assistance. IEEE Robot. Autom. Lett..

[69] Myseismic Powered Clothing (2023). Myseismic. https://www.myseismic.com.

[70] Li J.M., Molinaro D.D., King A.S., Mazumdar A., Young A.J. (2022). Design and validation of a cable-driven asymmetric back exosuit. IEEE Trans. Robot..

[71] Zhu K., Sharma B., Phan P.T., Davies J., Thai M.T., Hoang T.T., Nguyen C.C., Ji A., Nicotra E., Lovell N.H. Development of a Smart Textile-Driven Soft Spine Exosuit for Lifting Tasks in Industrial Applications. Proceedings of the IEEE/SICE International Symposium on System Integration (SII).

[72] Kim J.I., Choi J., Kim J., Song J., Park J., Park Y.-L. (2024). Bilateral Back Extensor Exosuit for multidimensional assistance and prevention of spinal injuries. Sci. Robot.

[73] Lazzaroni M., Toxiri S., Caldwell D.G., Anastasi S., Monica L., De Momi E., Ortiz J. Acceleration-based Assistive Strategy to Control a Back-support Exoskeleton for Load Handling: Preliminary Evaluation. Proceedings of the IEEE 16th International Conference on Rehabilitation Robotics (ICORR).

[74] Barsomian C., Eswaran N.B., Pesenti M., Gandolla M., Braghin F., Carpanzano E., Roveda L. (2024). Dynamic characterization and control of a back-support exoskeleton 3D-printed cycloidal actuator. CIRP Ann..

[75] Matijevich E.S., Volgyesi P., Zelik K.E. (2021). A Promising Wearable Solution for the Practical and Accurate Monitoring of Low Back Loading in Manual Material Handling. Sensors.

[76] Jung H., Seo J., Seo K., Kim D., Park S. (2021). Detection of Muscle Activation during Resistance Training Using Infrared Thermal Imaging. Sensors.

[77] Baker W.B., Parthasarathy A.B., Busch D.R., Mesquita R.C., Greenberg J.H., Yodh A.G. (2014). Modified Beer-Lambert law for blood flow. Biomed. Opt. Express.

[78] Sikora M., Paszkiel S. (2019). Muscle activity measurement using visible light and infrared. FAC-PapersOnLine.

[79] Hodges P.W., Pengel L.H., Herbert R.D., Gandevia S.C. (2003). Measurement of muscle contraction with ultrasound imaging. Muscle Nerve.

[80] Yang X., Chen Z., Hettiarachchi N., Yan J., Liu H. (2021). A Wearable Ultrasound System for Sensing Muscular Morphological Deformations. IEEE Trans. Syst. Man Cybern. Syst..

[81] Tarata M.T. (2003). Mechanomyography versus Electromyography, in monitoring the muscular fatigue. Biomed. Eng. Online.

[82] Chudecka M., Lubkowska A. (2010). Temperature changes of selected body’s surfaces of handball players in the course of training estimated by thermovision, and the study of the impact of physiological and morphological factors on the skin temperature. J. Therm. Biol..

[83] Hildebrandt C., Zeilberger K., John Ring E.F., Raschner C. (2012). The Application of Medical Infrared Thermography in Sports Medicine. An International Perspective on Topics in Sports Medicine and Sports Injury.

[84] Rodriguez-Sanz D., Losa-Iglesias M.E., Becerro-De-Bengoa-Vallejo R., Dorgham H.A.A., Benito-De-Pedro M., San-Antolín M., Mazoteras-Pardo V., Calvo-Lobo C. (2019). Thermography related to electromyography in runners with functional equinus condition after running. Phys. Ther. Sport.

[85] Perpetuini D., Formenti D., Cardone D., Trecroci A., Rossi A., Di Credico A., Merati G., Alberti G., Di Baldassarre A., Merla A. (2023). Can Data-Driven Supervised Machine Learning Approaches Applied to Infrared Thermal Imaging Data Estimate Muscular Activity and Fatigue?. Sensors.

[86] Xiao Z.G., Menon C. (2019). A Review of Force Myography Research and Development. Sensors.

[87] Sherif O., Bassuoni M.M., Mehrez O. (2024). A survey on the state of the art of force myography technique (FMG): Analysis and assessment. Med. Biol. Eng. Comput..

[88] Delva M.L., Lajoie K., Khoshnam M., Menon C. (2020). Wrist-worn wearables based on force myography: On the significance of user anthropometry. Biomed. Eng. Online.

[89] Gantenbein J., Ahmadizadeh C., Heeb O., Lambercy O., Menon C. (2023). Feasibility of force myography for the direct control of an assistive robotic hand orthosis in non-impaired individuals. J. Neuroeng. Rehabil..

[90] Rehman M.U., Shah K., Haq I.U., Iqbal S., Ismail M.A. (2023). A Wearable Force Myography-Based Armband for Recognition of Upper Limb Gestures. Sensors.

[91] von Arx M., Liechti M., Connolly L., Bangerter C., Meier M.L., Schmid S. (2021). From Stoop to Squat: A Comprehensive Analysis of Lumbar Loading Among Different Lifting Styles. Front. Bioeng. Biotechnol..

[92] Antsaklis P.J., Passino K.M. (1993). (Eds.). An Introduction to Intelligent and Autonomous Control.

[93] Radosavovic I., Xiao T., Zhang B., Darrell T., Jitendra Malik J., Sreenath K. (2023). Real-World Humanoid Locomotion with Reinforcement Learning. arXiv.

[94] Belo J.P.R., Romero R.A.F. A Social Human-Robot Interaction Simulator for Reinforcement Learning Systems, 2021. Proceedings of the 20th International Conference on Advanced Robotics (ICAR).

[95] Yan B., Zhu H., Li X., Li X. (2025). Reinforcement Learning Based Soccer Kicking for Humanoid Robots. Proceedings of the 2nd International Conference on the Frontiers of Robotics and Software Engineering (FRSE 2024).

[96] Muijzer-Witteveen H., Sibum N., van Dijsseldonk R., Keijsers N., van Asseldonk E. (2018). Questionnaire results of user experiences with wearable exoskeletons and their preferences for sensory feedback. J. Neuroeng. Rehabil..

[97] Ingraham K.A., Tucker M., Ames A.D., Rouse E.J., Shepherd M.K. (2023). Leveraging user preference in the design and evaluation of lower-limb exoskeletons and prostheses. Curr. Opin. Biomed. Eng..

[98] Pesenti M., Antonietti A., Gandolla M., Pedrocchi A. (2021). Towards a Functional Performance Validation Standard for Industrial Low-Back Exoskeletons: State of the Art Review. Sensors.

